# “A comparative analysis of risk factors of malaria” case study Gisagara and Bugesera District of Rwanda. RDHS 2014/2015. A retrospective study

**DOI:** 10.1186/s12889-023-15104-0

**Published:** 2023-01-25

**Authors:** Emmanuel Kubana, Athanase Munyaneza, Steven Sande, Felix Nduhuye, Jean Bosco Karangwa, David Mwesigye, Emmanuel Ndagijimana, Samuel Habimana, Cyprien Munyanshongore

**Affiliations:** College of Medicine & Health Sciences, University of Rwanda, Kigali, Rwanda

**Keywords:** CHWs, ICCM IRS, LLITN, Malaria, Reduced logistic regression, RDHS 2014/2015, Rwanda

## Abstract

**Background:**

Malaria is a public health concern worldwide. A figure of 3.2 billion people is at risk of malaria a report of World Health Organization in 2013. A proportion of 89 and 91 cases of malaria reported during 2015 were respectively attributed to malaria cases and malaria deaths in Sub-Saharan Africa. Rwanda is among the Sub-Saharan Africa located in East Africa. The several reports indicate that from 2001 to 2011, malaria cases increased considerably especially in Eastern and Southern Province with five million cases. The affected districts included Bugesera in the Eastern and Gisagara in the Southern Province of Rwanda with a share of 41% of the country prevalence in 2014 and during 2017–2018 a figure of 11 deaths was attributed to malaria and both Gisagara and Bugesera Districts were the high burdened.

**Methodology:**

The RDHS 2014–2015 data was used for the study and a cross-sectional survey was used in which two clusters were considered both Gisagara and Bugesera Districts in the Southern and Eastern Province of Rwanda. Bivariate analysis was used to determine the significant predictors with malaria and reduced logistic regression model was used.

**Results:**

The results of the study show that not having mosquito bed nets for sleeping is 0.264 times less likely of having malaria than those who have mosquito bed nets in Gisagara District. In Bugesera District, living in low altitude is 2.768 times more likely associated with the risk of getting malaria than living in high altitude.

**Conclusion:**

The results of the study concluded that environmental and geographical factor such as low altitude is the risk factor associated with malaria than the high altitude in Bugesera District. While not having mosquito bed nets for sleeping is the protective factor for malaria than those who have it in Gisagara District. On the other hand, socio-economic and demographic characteristics do not have any effect with malaria on the results of the study.

## Background

Malaria is still a public health concern worldwide [1, 2]. Estimates of 3.2 billion people worldwide are reported to be at risk of malaria by The World Health Organization in 2013 (WHO). During 2015, 89% of malaria cases and 91% of malaria deaths of the global burden of malaria were attributed to Sub-Saharan Africa [3, 4]. Although Algeria, one of the African countries that requested the free-malaria certificate from World Health Organization [5], data of 2017 indicated that this country to be the most with 448 imported malaria cases with 448 cases. These cases are more prevalent in the area of the Southern Province of Tamanrasset bordering both Mali and Niger considered as the endemic area attributed 81% of the imported malaria [5].

In East-Africa, Uganda ranked at the third position of total malaria cases among African countries and Mauritius is the only Sub-Saharan country to achieve malaria elimination target [6, 7]. The reports revealed that the risk factors attributed to the increase of malaria in Rwanda are Substandard Long Lasting Insecticide treated Nets (LLINs), Climatic data anomalies such as rainfall and changes in ambient temperature and Insecticide resistance through documented emerging parathyroid resistance all of these factors contributed high burden of malaria in Rwanda [3, 4].

Between 2001 and 2015, policies for malaria control interventions in Sub-Saharan Africa countries highlighted that Insecticide-treated nets (LLITNs) and Indoor residual spraying (IRS) contributed for 70% of the 943 million in reduction of malaria cases [4].

Recently, Rwanda is among sub-Sahara African countries in which the prevalence of malaria is high [8]. During the year 2017–2018, reports revealed a record of over 11 cases of deaths attributed to malaria where Gisagara and Bugesera were among the highest with cases of deaths due to malaria [9, 10].

In Rwanda, although from 2002 to 2011, health facilities reported more than five million cases [3, 6] from 2005 to 2012, there was a reduction of about 86% and 74% in malaria incidence and mortality respectively [3, 6], recent data indicates that Rwanda is among the SSA with the highest malaria prevalence [8]. In 2013, one million cases of malaria were reported and high prevalence was observed in rural areas, with the most affected districts being Bugesera, Kamonyi, and Gisagara [3, 4].

.

During the year 2014, reports showed that the high burden of malaria presented especially in the districts located in the Eastern Province represent a prevalence of 41% of total cases and these are: Kirehe, Ngoma, Bugesera, Kayonza, Rwamagana. The Ruhuha Sector in Rwanda is one area burdened by malaria prevalence, with an estimated slide positivity rate of 5% [10, 11]. The area is located in Bugesera District of the Eastern Province, household survey results conducted in Ruhuha classify it as hypo-endemic for malaria, with cases clustered around marshlands. Individuals from households with high socioeconomic status have a lower risk of contracting malaria [3].

In the Southern Province, the districts with a high disease burden represent a prevalence of 38% of total malaria cases. These are: Gisagara, Nyanza, Huye, Kamonyi, Ruhango, Muhanga [9, 10].

Besides the fact that Bugesera and Gisagara districts share almost the same proportion of malaria cases in the country, they border Burundi, another sub-Saharan African country considered endemic for malaria, and imported cases would also contribute significantly to the observed cases. To our knowledge, there is little knowledge of other household or individual characteristics that might contribute to the increase in observed malaria cases in these two districts. This study aimed to determine the prevalence and risk factors associated with malaria among the general Rwandan population in Gisagara and Bugesera districts in Rwanda.

## Methods and materials

### Study design, setting and population

This was a cross-sectional survey analyzing secondary data from the Rwanda Demographic and Health Survey (RDHS) 2014/2015 to determine the prevalence and risk factors of malaria in Bugesera and Gisagara districts in Rwanda.In Rwanda, the health system includes the followings main levels: Community, Health Center, District Hospital, Provincial and Referal (Teaching Hospitals). Community Health Workers (CHWs) provide household level health education, case finding for acute and chronic illness, Integrated Community Case Management, ICCM (including diagnosis and treatment of pneumonia, diarrhea, and malaria), female contraception, and linkage to health facilities for prenatal care, deliveries, and other medical services. Each of the health centers serve a catchment area of approximately 20,000–30,000 people and are staffed by general nurses who provide basic diagnostics, outpatient acute services, family planning, prenatal care, and routine deliveries [12, 13]. The target population was all people in Bugesera and Gisagara districts in Rwanda who participated in a 2014/2015 survey.

### Sample size calculation and sampling techniques

The sample size is the total population filled the questionnaire of the Rwanda Demographic Health Survey of 2014/2015 in Gisagara and Bugesera Districts.

The study used a RDHS data of 2014/2015 whereby all population stayed in their home in the evening preceding the interview were considered as the sample size of the study.

A representative sample of 13,497 was selected for the 2014/2015 RDHS. The sample was selected in two stages.

The first stage consisted of 492 selected villages with probability proportional to the village size (also known as clusters or enumeration areas). The second phase of sampling consisted of a complete mapping and listing of all households in the selected villages. The obtained lists of households served as the sampling frame for the second stage of sample selection. Households were systematically selected from those lists for participation in the survey [14].The 2014/2015 RDHS was the sixth national health survey for which data collection was conducted from November 9, 2014 to April 8, 2015. All of the 492 clusters selected for the sample were surveyed. A representative’s sample size of 741 was selected in both Districts (Bugesera and Gisagara) as the households who accepted to respond the questions regarding malaria in 2014/2015 RDHS questionnaire, with a representative total sample size of 387 in Gisagara District and 354 in Bugesera District among the whole population of 12,699 who completed the household questionnaire in whole country for the 2014/2015 RDHS.

### Data collection procedures

Rwanda Demographic Health Survey of November 9th/2014 to April 8th/2015 was used for secondary data analysis. The first step was the registration and application to RDHS datasets. The researcher made the request to use the datasets on June 11th 2019 and was approved on June 11th 2019 of both Gisagara and Bugesera Districts.

The researcher agreed the terms and conditions in using DHS data set such that it is not allowed to make any effort for identifying respondents, households addresses or sample communities in which the Institutional Review Board approved procedures for DHS public use datasets. The second step was to download the original datasets from RDHS 2014/2015, Datasets of Gisagara and Bugesera Districts was extracted from the RDHS 2014/15 datasets from the datasets of Rwanda and used for obtaining and describing the comparative risk factors of malaria and characteristics of the population within the selected areas of the study. Therefore, the original data to compute the malaria prevalence were derived from the Malaria section of the Questionnaire used in DHS 2014/2015.

## Materials

### Study tools

The data used to compute the malaria cases were derived from the malaria history Questionnaire used in RDHS 2014/2015. Data will be analyzed using STATA version 13.0.

### Ethical considerations

Confidentiality: The personal details of participants were not requested to the RDHS team when applying for the dataset. The researchers that participated in the RDHS 2014/2015 will not participate in the present study and we will not make any efforts to identify the study participants. The study data will be analyzed anonymously.

#### Informed consent/Assent form

This study had no direct contact with humans or with any identifiable information/parts of human beings. The researcher found no need for an informed consent for participation; hence no informed consent will be needed.

## Results

### Socio-economic and demographic characteristics of the study population

The results from Table 1 printed out.

**Table 1:** Socio-economic and demographic characteristics of the study population

		Gisagara (N = 387)		Bugesera (N = 354)	
Variables		Frequency	Percent	Frequency	Percent
Sex	Male	263	67.96	292	82.49
	Female	124	32.04	62	17.51
	< 20	3	0.78	0	0
	21–40	172	44.44	240	67.8
Age (in years)	41–60	184	47.55	98	27.68
	61–80	24	6.2	16	4.52
	> 80	4	1.03	0	0
	No education, preschool	186	48.06	166	46.89
	Primary	174	44.96	142	40.11
Education	Secondary	26	6.72	43	12.15
	Higher	1	0.26	3	0.85
	Never married	95	40.43	62	29.81
	Married	81	34.47	72	34.62
Marital status	Living together	34	14.47	52	25
	Widowed	9	3.83	10	4.81
	Divorced	1	0.43	5	2.4
	Not living together	15	6.38	7	3.37
	Poorest	208	53.75	57	16.1
	Poorer	80	20.67	59	16.67
Wealth index	Middle	37	9.56	90	25.42
	Richer	35	9.04	83	23.45
	Richest	27	6.98	65	18.36
Place of cooking	In the house	242	62.53	32	9.04
	In a separate building	122	31.52	247	69.77
	Outdoors	23	5.94	75	21.19
Household has separate room used as Kitchen	No	51	21.25	13	40.63
	Yes	189	78.75	19	59.38
Owns land usable for Agriculture	No	107	27.65	113	31.92
	Yes	280	72.35	241	68.08
	0	134	38.73	138	38.98
Agriculture land size (in hectares)	1–5	45	13.01	45	12.71
	5–10	32	9.25	39	11.02
	Above 10	135	39.02	132	37.29
Owns livestock herds or farm animals	No	210	54.26	139	39.27
	Yes	177	45.74	215	60.73
Having Bank Account	No	241	62.27	161	45.48
	Yes	146	37.73	193	54.52

The majority of interviewed head of households did not have any education level at least 48.06% and 46.89 in Gisagara and Bugesera Districts respectively attended either primary school only, whereas **44.96%** and **40.11%** attended at least primary school in Gisagara and Bugesera District respectively. Only **34.47%** and **34.62%** of heads of household were married in Gisagara and Bugesera District respectively. The results showed that the majority were the heads of household who were never married with a rate of **40.43%** and **29.81%** in Gisagara and Bugesera District respectively.

Among all interviewed heads of Household in Gisagara District, 53.75% were poorest while 20.67% were poor. Contrary, in Bugesera District the majority of interviewed heads of household were in middle and richer category with a rate of 25.42% and 23.45% respectively.

The higher level of poverty found while analyzing this data may higher related with the fact that the majority of households especially those visited in Gisagara District didn’t have enough agriculture land size, owns their livestock’s nor having the ability to save some money on bank account. As it was found, the majority of households visited in Gisagara District cook their foods in the sleeping house 62.53% comparatively to those of Bugesera District whereas the majority 69.77% preparing the foods in the house separated with the main sleeping houses. The cooking place maybe the greatest factor for malaria infection among the household’s members of Bugesera District, as the major of the them 69.77% move from one house to another for the food cooking, and some of them 21.19% using outdoor for the food cooking.

### Prevalence of malaria in Bugesera and Gisagara Districts

The results of the study from Table 2 show that in Gisagara District the level of malaria prevalence is low compared to the level of malaria prevalence in Bugesera District. The figures of malaria prevalence are as follow 10.08% and 12.71% in Gisagara and Bugesera Districts respectively.

**Table 2:** Prevalence of malaria in Bugesera and Gisagara Districts

	Gisagara District		Bugesera District	
Results of Malaria tests	Frequency	Percentages (%)	Frequency	Percentages (%)
Negatives	348	89.92	309	87.29
Positives	39	**10.08**	45	**12.71**

### Geographical and environmental status of Gisagara and Bugesera Districts

The results from this survey printed out that majority of interviewed households living in rural areas at level of 86.05% and 85.03% in Gisagara and Bugesera Districts respectively, and 13.95% and 14.97% who lived in urban, this may the reason malaria found much more in those areas of study. There were still a higher number of people who took long journey within the year of 2014–2015 in order to reach the hospital in those areas of study. A higher number 46.40% in Gisagara District went from home up to nearly District Hospital or Health Center for interval length of 200-500 m while in Bugesera District a higher number 49,68% of interviewed households went for length above 500 m in order to reach a hospital or health center. The majority of the households in Bugesera District were located in low altitude, whereas all of them 100% living in different agro-ecological zone with altitude under 1500 m. Contrary to the households of Gisagara District, majority of those lived on higher altitudes. According to geographical location, these two districts, are located near the long rivers (Akanyaru and Akagera rivers) of country pass through them before reaching abroad and became Nil. These two Districts were covered with several marshlands and swamps that were the suitable areas for the development of mosquitos. This is maybe also the reason by which Gisagara and Bugesera were two Districts with higher number of positive results tests of Malaria in the country. According to the results in Table 3;

**Table 3:** Geographical and environmental status of Gisagara and Bugesera Districts

		Gisagara (N = 387)		Bugesera (N = 354)	
Variables		Frequency	Percent	Frequency	Percent
Having a radio	No	198	51.16	168	47.46
	Yes	189	48.84	186	52.54
Having a Television	No	372	96.12	302	85.31
	Yes	15	3.88	52	14.69
Having a mobile	No	239	61.76	90	25.42
	Yes	148	38.24	264	74.58
Having mosquito bed net	No	42	10.85	42	11.86
	Yes	345	89.15	312	88.14
Type of Mosquito bed net used	Did not sleep under mosquito net	125	32.3	84	23.73
	Only treated Mosquito net	262	67.7	270	76.27
Person sleeping under the treated net	No	125	32.3	84	23.73
	Yes	262	67.7	270	76.27
Covered by health insurance	No	130	33.68	103	29.1
	Yes	256	66.32	251	70.9
HH Residence	Urban	54	13.95	53	14.97
	Rural	333	86.05	302	85.03
Distance from house (in meters)	> 200	109	29.07	28	9.03
	200–500	174	46.4	128	41.29
	< 500	92	24.53	154	49.68
	1301–1400	17	4.39	197	55.65
	1401–1500	71	18.35	157	44.35
Altitudes ( in meters)	1501–1600	221	57.11	0	0
	1601–1700	51	13.18	0	0
	1701–1800	27	6.98	0	0

## Bivariate analysis

### Bivariate analysis of malaria and socio-economic and demographic characteristics of the study population

The results of the bivariate analysis of Malaria and socio-economic and demographic characteristics of the study population **from** Table 4 show that in Gisagara District, two variables including owns livestock herds or farm animals and having bank account were statistically significant with Malaria at a P-value of 0.02 and 0.04 respectively. For Bugesera District, none variable was statistically significant with malaria.

**Table 4:** Bivariate analysis of Malaria and socio-economic and demographic characteristics of the study population

Results of Malaria tests in percentage							
		**Gisagara (N = 387)**			**Bugesera (N = 354)**		
Variables		Positives N = 39	Negatives N = 348	P-Value	Positives N = 45	Negatives N = 309	P-Value
Sex	Male	64.10(25)	68.39(238)	0.586	88.89 (40)	81.55(252)	0.226
	Female	35.90 (14)	31.61(110)		11.11 (5)	18.45 (57)	
	< 20	2.56 (1)	0.57 (2)		0.00 (0)	0.00 (0)	
Age (in years)	21–40	28.21 (11)	46.26(161)	0.128	64.44 (29)	68.28(211)	0.073
	41–60	58.97(23)	46.26 (161)		24.44 (11)	28.16 (87)	
	61–80	10.26(4)	5.75 (20)		11.11 (5)	3.56 (11)	
	> 80	0.00 (0)	1.15 (4)		0.00 (0)	0.00(0)	
	no education, preschool	56.41 (22)	47.13 (164)		62.22 (28)	44.66 (138)	
Education	Primary	35.90(14)	45.98(160)	0.661	33.33 (15)	41.10 (127)	0.105
	Secondary	7.69 (3)	6.61(23)		4.44(2)	13.27 (41)	
	Higher	0.00(0)	0.29(1)		0.00 (0)	0.97(3)	
	Never Married	72.22 (13)	37.79 (82)		47.06 (8)	28.27(54)	
Marital Status	Married	5.56 (1)	36.87(80)		23.53 (4)	35.60 (68)	
	Living together	16.67 (3)	14.29 (31)		29.41(5)	24.61 (47)	
	Widowed	0.00 (0)	4.15 (9)	0.062	0.00 (0)	5.24(10)	0.466
	Divorced	0.00 (0)	0.46 (1)		0.00 (0)	2.62 (5)	
	Not living together	5.56(1)	6.45 (14)		0.00 (0)	3.66 (7)	
	Poorest	74.36 (29)	51.44 (179)		28.89 (13)	14.24 (44)	
Weath Index	Poorer	15.38(6)	21.26 (74)		15.56 (7)	16.83 (52)	
	Middle	0.00 (0)	10.63 (37)	0.058	26.67 (12)	25.24 (78)	0.112
	Richer	5.13 (2)	9.48 (33)		17.78 (8)	24.27 (75)	
	Richest	5.13 (2)	7.18(25)		11.11 (5)	19.42 (60)	
	In the house	66.67 (26)	62.07 (216)		13.33 (6)	8.41 (26)	
Cooking areas	In separate building	28.21 (11)	31.90 (111)	0.853	60.00 (27)	71.20 (220)	0.288
	Outdoors	5.13 (2)	6.03 (21)		26.67 (12)	20.39 (63)	
House has separate room used as Kitchen	No	19.23 (5)	21.50 (46)	0.79	33.33(2)	42.31 (11)	0.687
	Yes	80.77(21)	78.50(168)		66.67(4)	57.69 (15)	
Owns land used for agriculture	No	33.33 (13)	27.01 (94)	0.403	31.11 (14)	32.04 (99)	0.901
	Yes	66.67 (26)	72.99 (254)		68.89 (31)	67.96(210)	
Agriculture land Size(in hectares)	0	42.86 (15)	38.26 (119)		40.00(18)	40.00 (120)	
	1-5	5.71(2)	13.83 (43)		20.00(9)	11.65 (36)	
	5-10	2.86 (1)	9.87 (31)	0.222	22.22 (1)	12.30 (38)	0.122
	Above 10	48.57(17)	37.94 (118)		37.78(17)	37.22(115)	
Owns livestock Herds or farm animals	No	71.79(28)	52.30 (182)	0.02**	33.33(15)	40.13 (124)	0.383
	Yes	28.215 (11)	47.705(166)		66.67(30)	59.87 (185)	
Having Bank Account	No	76.92 (30)	60.63 (211)	0.047**	51.11 (23)	44.66 (139)	0.417
	Yes	23.08 (9)	39.37 (137)		48.89(22)	55.34 (171)	

### Bivariate analysis of malaria and geographical and environmental status of Gisagara and Bugesera districts

The results from Table 5 show that for Gisagara District variables such as having radio, having mobile phone, having mosquito bed net, type of mosquito bed net used, person sleeping under the treated net, covered by health insurance were statistically significant.

For Bugesera District, three variables were statistically significant. Those variables include; type of mosquito bed net used with a p-value of 0.001 in Gisagara district, person sleeping under the treated net with a p-value of 0.001 in Gisagara district, Having mosquito bet net with a p-value of 0.000 in Gisagara district, covered by health insurance with a p-value of 0.000 in Gisagara districtand altitudes with a p-value of 0.004 in Bugesera District.

**Table 5:** Bivariate analysis of malaria and Geographical and environmental status of Gisagara and Bugesera Districts

Results of malaria tests in percentages							
			Gisagara (N = 387)			Bugesera (N = 354)	
Variables		Positives (N = 39)	Negatives (N = 348)	P-Value	Positives (N = 45)	Negatives (N = 309	P-Value
Having a radio	No	71.79 (28)	48.85 (170)	0.007***	48.89 (22)	47.25 (146)	0.837
	Yes	28.21 (11)	51.15 (178)		51.11 (23)	52.75 (163)	
Having a Television	No	97.44 (38)	95.98 (334)	0.654	91.11 (41)	84.47 (261)	0.239
	Yes	2.56 (1)	4.02 (14)		8.89 (4)	15.53 (48)	
Having mobile Phone	No	76.92 (30)	60.06 (209)	0.04**	28.89 (13)	24.92 (77)	0.568
	Yes	23.08 (9)	39.94 (139)		71.11 (32)	75.08 (232)	
Having mosquito bed net	No	30.77 (12)	8.62 (30)	0.000***	20.00 (9)	10.68 (33)	0.071
	Yes	69.23 (27)	91.38 (318)		80.00 (36)	89.32 (276)	
Type of Mosquito bed net used	Did not sleep under mosquito net	56.41 (22)	29.60 (103)	0.001***	35.56 (16)	22.01 (68)	0.046**
	Only treated Mosquito net 43.59 (17)		70.40 (245)		64.44 (29)	77.99 (241)	
Person sleeping under the treated net	No	56.41 (22)	29.60 (103)	0.001***	35.56 (16)	22.01 (68)	0.046**
	Yes	43.59 (17)	70.40 (245)		64.44 (29)	77.99 (241)	
Covered by health insurance	No	64.10 (25)	30.26 (105)	0.000***	37.78 (17)	27.83 (86)	0.17
	Yes	39.50 (14)	69.74 (242)		62.22 (28)	72.17 (223)	
HH Residence	Urban	20.51 (8)	13.22 (46)	0.213	13.33 (6)	15.21 (47)	0.742
	Rural	79.49 (31)	86.78 (302)		86.67 (39)	84.79 (262)	
Distance from House ( in meters)	> 200	18.42 (7)	30.27 (102)		2.27 (1)	10.15 (27)	
	200–500	47.37 (18)	46.29 (156)	0.195	54.55 (24)	39.10 (104)	0.074
	< 500	34.21 (13)	23.44 (79)		43.18 (19)	50.75 (135)	
Altitudes ( in meters)	1301–1400	7.69 (3)	4.02 (14)		75.56 (34)	52.75 (163)	
	1401–1500 20.51 (8)		18.10 (63)		24.44 (11)	47.25 (146)	
	1501 − 1500	66.67 (26)	56.03 (195)	0.147	0.00 (0)	0.00 (0)	0.004***
	1501–1600	2.56 (1)	14.37 (50)		0.00 (0)	0.00 (0)	
	1601–1700	2.56 (1)	7.47 (26)		0.00 (0)	0.00 (0)	

### Multivariate analysis of malaria in Gisagara and Bugesera Districts

The results of Table 6 from the reduced logistic regression model used the significant variables with malaria in both Gisagara and Bugesera Districts. For Gisagara District, the bivariate analysis of the factors like having radio, having mosquito bed net for sleep, having mobile phone, type of mosquito bed net, person sleeping under treated net, having health insurance, owns livestock herds or farm animals, having bank account were included in reduced model. In Bugesera District, variables such as type of mosquito bed net, person sleeping under the treated net and altitudes were considered for reduced regression model.

According to the multivariate analysis only not having mosquito bed nets for sleeping with OR = 0.264 CI = [0.118, 0.593], it has an association with malaria in Gisagara District. Therefore, in Gisagara District not having mosquito bed nets for sleeping is 0.264 times less likely of having malaria than those who have mosquito bed nets.

The results of multivariate analysis in Bugesera District show one variable with a significant level. The explanatory variable such low as altitude with OR 2.768 CI [1.353–5.662] is 2.768 times more likely to have malaria than those of high altitude.

**Table 6:** Multivariate Analysis of Malaria in Gisagara and Bugesera Districts

Gisagara District				Bugesera District		
Results of Malaria tests	OR	CI at 95%	Pv	OR	CI at 95%	pv
Having a radio	1			-		
No	1	-	-	-	-	-
Having mosquito bed nets for sleeping	1			-		
No	0.264	0.118–0.593	0.001***	-	-	-
Having a mobile phone	1			-		
No	1	-	-	-	-	-
Owns livestock	1					
Yes	0.630	0.277–1.431	0.27	-	-	-
Having a bank account	1			-		
Yes	0.697	0.293–1.656	0.414	-	-	-
Using mosquito bed net last night	1			1		
Both treated (itn) and Untreated nets	1	-	-	1		
Person slept under even treated net	1			1		
N0	1			1		
Covered by health insurance	1			-		
No	1	-	-	-	-	-
Altitudes (1401–1500)	-			1		
1301–1400	-	-	-	2.768	1.353–5.662	0.005***
1501–1600	-	-	-	-	-	-
1601–1700	-	-	-	-	-	-
1701–1800	-	-	-	-	-	-

The sign in table means: *** P-value < 0.01%, ** P-value < 0.05 and * P-value < 0.1%. Test differences for vegetable farmers characteristics through independents t-test and chi-square.

## Discussion

### Prevalence of malaria in Gisagara and Bugesera districts

According to the 2010 RDHS, malaria prevalence has decreased from 2.6% to 2008 to 1.4% in 2010 in children under 5 years and a decline from 1.4% to 2008 to 0.7% in 2010 of malaria prevalence in pregnant women [15]. In this study it was proven that among all surveyed households in Gisagara and Bugesera Districts, the prevalence rate of malaria was 10.08% (39/387) and 12.71% (45/354) in Gisagara and Bugesera Districts respectively. The higher rate in Bugesera District comparatively to Gisagara with the rate of 88.89% in male and 11.11% in female in Bugesera District compared to 64.10% in male and 35.90% in female in Gisagara District among all infected by malaria in the study areas. According to the conducted study in Kola Diba, North Gondar, Ethiopia of ten year trend analysis of malaria prevalence, the results showed that the majority of men were infected by malaria with the infection rate of 52.6% in males and 47.3 in females, the same as the results of our study [16].

### Associated factors

The analysis results show that, the majority of households lived on altitudes less than 1400 m were much more exposed to malaria disease more than those who were living on elevation greater than 1400 m in Bugesera District. The more infected by malaria in Bugesera District were 52.75%, located on elevation less than 1400 m while 47.25% people infected by malaria were living on elevation above 1400 m.In Gisagara District, majority of the patients infected by malaria, their cluster were on altitudes less than 1500 m. The altitude in Gisagara District was not statistically significant, while in Bugesera District, altitude was statistically significant with malaria proven by the P-value of 0.005 which was less than P-value at the statistically significant level. The same results conducted in Uganda, on prevalence and risk factors of malaria in Uganda, proved that the majority of the sampled households 92.5% were in clusters with altitudes ranging between 1000 and 1500 m. A very small portion of the households 7.4% was in clusters with altitudes higher than 1500 m, where malaria transmission is lower [17].

According to the results of multivariate analysis in Gisagara District, it showed that, visited and interviewed households who had not mosquito bed nets for sleeping were 0.264 times less likely to be infected by malaria if compare with those who had mosquito bed nets for sleeping with OR = 0.264, 95% CI=[0.118, 0.593]. The results on individual, household and environmental risk factors for malaria infection conducted in Ethiopia showed that sleeping or not sleeping under LLNs did not statistically significant with malaria.

This protective factor in Gisagara District may be associated in others factors not listed in this study like prevention using indoor residual spraying (IRS), cutting of bush around the houses, sleeping times, having mosquito bed nets and not used it correctly, having mosquito bed nets and those mosquito do not meet the standards( not treated, having holes around, not cover the all beds), etc. [18].

### Study limitations

A comparative analysis of risk factors of malaria in both Gisagara and Bugesera Districts of Rwanda was associated with the following limitations:

As the study was a Cross-sectional survey using DHS data, some independents variables were missing.

Despite this limitation, furthermore, the objectives of the study were reached.

## Conclusion and recommendations

The results of our study showed a prevalence of 10.08% and 12.71% in Gisagara and Bugesera Districts respectively which was high in Bugesera District than in Gisagara District.

The results of environmental and geographical status in both Districts such altitude was statistically significant with malaria in Bugesera District and not having mosquito bed nets for sleeping was the protective factor with malaria than those who have mosquito bed nets in Gisagara District.

Based on the results of our research which show high prevalence rate of malaria in Bugesera District than in Gisagara District and based on the associated risk factors such not having mosquito bed nets for sleeping which resulted as a protective predictor in Gisagara District and in household lived in low altitude resulted as an associated factor with malaria than those who lived in high altitude in Bugesera District.

The recommendations are as follow: The policymakers, the local leader, stakeholders and the all community to take action, engagement and participation in implementing the policies elaborated for malaria prevention and eradication. To provide appropriate prevention measures such as mosquito bet net in risk zone areas and to regular check their status.

To elaborate different policies and implementation strategies by combination of community participation during the process of policy formulation for malaria.

## Data Availability

Rwanda Demographic Health Survey of November 9th/2014 to April 8th/2015 was used for secondary data analysis. The first step was the registration and application to RDHS datasets. The researcher made the request to use the datasets on June 11th 2019 and was approved on June 11th 2019 of both Gisagara and Bugesera Districts. The researcher agreed the terms and conditions in using DHS data set such that it is not allowed to make any effort for identifying respondents, households addresses or sample communities in which the Institutional Review Board approved procedures for DHS public use datasets. The data used to compute the malaria cases were derived from the malaria history Questionnaire used in RDHS 2014/2015. Data was analyzed using STATA version 13.0. The direct web link to the database is: https://www.dhsprogram.com/data/dataset_admin/login_main.cfm.
